# Loss of NOS1 expression in high-grade renal cell carcinoma associated with a shift of NO signalling

**DOI:** 10.1038/sj.bjc.6601809

**Published:** 2004-05-11

**Authors:** K Renaudin, M G Denis, G Karam, G Vallette, F Buzelin, C L Laboisse, A Jarry

**Affiliations:** 1 Pathology Department, CHU Hôtel Dieu, 30 Boulevard Jean Monnet, 44093 Nantes Cedex 1, France; 2 INSERM U539, Faculty of Medicine, 1 Rue Gaston Veil, 44035 Nantes Cedex1, France; 3 Urology Department, CHU Hôtel Dieu, Place Alexis Ricordeau, 44093 Nantes Cedex 1, France

**Keywords:** NOS, human kidney, renal cell carcinoma, soluble guanylate cyclase, protein nitration, NO signalling

## Abstract

In normal human kidney, NOS1 and soluble guanylate cyclase (sGC) are expressed in tubular epithelial cells, suggesting a physiological autocrine NO signalling pathway. Therefore, we investigated both NOS1 and sGC expressions in benign and malignant renal tumours. In addition, we examined the pattern of protein tyrosine nitration in normal and tumour tissue. NOS1 expression and activity were found to be downregulated, correlating with the tumour grade, as shown by immunohistochemistry, quantitative RT–PCR analysis, and histochemical detection of the NADPH-diaphorase activity of nitric oxide synthases (NOS). These results show that the autocrine NO signalling pathway is maintained in benign tumours and lost in malignant tumours. In contrast, sGC expression was maintained in renal tumours whatever the tumour type, a finding showing that tumour cells remain sensitive to the bioregulatory role of exogeneous NO^•^. Finally, the staining pattern of protein tyrosine nitration, assessed by immunohistochemistry, parallelled that of NOS1 expression in normal renal parenchyma and benign tumours, supporting the concept that protein nitration was accounted for by NOS1 activity. In contrast, in malignant tumours, protein tyrosine nitration was accounted for by the production of reactive nitrogen oxide species by the inflammatory infiltrate. Altogether, these findings argue for a pattern of NO signalling similar in normal kidney and benign renal tumours, whereas it is completely different in malignant renal tumours.

Nitric oxide synthases (NOS) are a family of enzymes that catalyse the synthesis of NO, a gaseous free radical involved in both physiological and pathological mechanisms depending on its concentration, cellular source and microenvironment (Nathan, 1992; Kone, 1997; Michel and Feron, 1997; Alderton *et al*, 2001). Three isoforms have been described, two ‘constitutive’, Ca^2+^-dependent isoforms, that is, NOS1 or neuronal NOS and NOS3 or endothelial NOS, and one ‘inducible’, Ca^2+^-independent isoform, NOS2 (for general reviews, see Michel and Feron, 1997; Alderton *et al*, 2001).

We have recently shown that most tubular epithelial cells along the human nephron strongly express NOS1, as well as soluble guanylate cyclase (sGC), the intracellular target of NO^•^, leading to an autocrine NO signalling pathway in physiological conditions (Jarry *et al*, 2003). Given that NO^•^ has an important bioregulatory role through its direct effects on sGC or by regulating the electron transport chain in mitochondria (Wink *et al*, 1996; Wink and Mitchell, 1998), one can wonder whether this autocrine signalling pathway is maintained or not in renal tumours. NOS1 is of particular interest since, besides synthesising NO^•^, it can also produce reactive oxygen species including superoxide ions (O_2_^−^) and hydrogen peroxide (H_2_O_2_) at low L-arginine concentrations (Xia *et al*, 1996). In addition, the combination of equimolar production of O_2_^−^ from several sources and NO^•^ forms peroxynitrite (ONOO^−^), an oxidising and nitrating agent leading to the nitration of protein tyrosine residues limited intracellularly within close proximity of its source (Ischiropoulos *et al*, 1992; Beckman *et al*, 1994; Jourd’heuil *et al*, 2001). Together, these findings strongly suggest that protein tyrosine nitration is a post-translational modification of proteins associated with NOS1 activity (Williams *et al*, 1998). This concept is in line with recent findings suggesting that ONOO^−^ via protein tyrosine nitration could have bioregulatory physiological roles, for example, modulation of Na+ K+ ATPase in rat kidney (Zhang *et al*, 2002). In pathological states, especially in inflammatory conditions, protein tyrosine nitration can result from complex mechanisms involving the formation of NO_2_^•^ via the oxidation of circulating nitrite NO_2_^−^ catalysed by peroxidase or haeme proteins, a process able to target even cells devoid of the endogeneous NO production machinery such as red blood cells (Ischiropoulos, 1998; Wink and Mitchell, 1998, Grzelak *et al*, 2001).

Several lines of investigation have pointed to a bioregulatory role associated with protein tyrosine nitration such as enzymatic regulation, for example, inactivation of manganese superoxide dismutase (MacMillan-Crow *et al*, 1996), cell proliferation and apoptosis (Williams *et al*, 1998; Brito *et al*, 1999). It is thus important to assess the extent of protein tyrosine nitration in normal and tumour tissues.

In this context, we were prompted to examine in benign and malignant renal tumours several issues, namely (i) the status of NOS1 expression in renal tumours of various grades of malignancy at the protein and mRNA levels; (ii) the persistence or not of an autocrine NO signalling pathway in renal tumours assessed by the immunohistochemical detection of sGC; and (iii) the pattern of protein tyrosine nitration in human normal kidney and in renal tumours, assessed by immunohistochemical detection of 3-nitrotyrosine. We used red blood cells in order to distinguish protein tyrosine nitration resulting either from an intracellular production of ONOO^−^ (negative staining of red blood cells) or from the production by the inflammatory infiltrate of reactive nitrogen species able to act at distance from their source (positive staining of red blood cells).

## MATERIALS AND METHODS

### Tissue specimens

Tumour tissue and normal tissue at distance from the tumour were collected from 32 patients undergoing nephrectomy for oncocytoma (six cases), chromophobe renal cell carcinoma (RCC) (four cases) and clear cell RCC (22 cases: five cases Führman grade I; seven cases Führman grade II; seven cases Führman grade III; three cases Führman grade IV). For each case, samples of tumour and normal tissue were snap frozen and stored in liquid nitrogen, and also fixed in formalin and embedded in paraffin. These samples were collected according to the guidelines of the French Ethics Committee for Research on Human Tissues.

### Immunohistochemistry

Immunohistochemistry was performed on 5 *μ*m formalin-fixed paraffin sections of different renal tumours with the polyclonal N-terminus NOS1, the sCG and the 3-nitrotyrosine antibodies, and on 5 *μ*m acetone-fixed cryostat sections for staining with the monoclonal C-terminus NOS1, the NOS2 and the NOS3 antibodies. The specificity and conditions of use of primary antibodies directed to NOS isoforms are listed in Table 1

**Table 1:** Antibodies used for immunohistochemistry

**Protein detected**	**Type of antibody source**	**Antigen (amino acids) C- or N-terminus**	**Immunohistochemistry: Frozen (F), paraffin (P)/dilution**
NOS1	Monoclonal	Synthetic human peptide	(F)/1 : 100
	Transduction Lab	C-terminus (1095–1289)	
			
NOS1	Polyclonal	Rat recombinant protein	(P)/1 : 200
	Zymed	N-terminus (last 200 aa)	
			
NOS2	Polyclonal	Synthetic human peptide	(F)/1 : 500
	Santa Cruz	C-terminus	
			
NOS3	Polyclonal	Synthetic human peptide	(F)/1 : 300
	Transduction Lab	C-terminus	
			
sGC	PolyclonalCayman	Synthetic human peptide from *α*1 and *β*1 subunits	(P)/1 : 300
			
3-NTY	Polyclonal	Nitrated KLH	(P)/1 : 300
	Upstate Biotechnology		

. First, endogenous biotin was blocked using a sequential avidin–biotin treatment (Biotin blocking system, Dako, Trappes, France). Then, immunohistochemistry was performed using a streptavidin–biotin method (Histostain Plus kit, LAB-SA detection system, Zymed, Cliniscience, Montrouge, France), according to the manufacturer's instructions. Aminoethyl carbazol (AEC) or diaminobenzidine (DAB) were used as chromogens. The sections were slightly counterstained with haematoxylin and mounted. Negative controls were performed by omitting the first antibody. To verify the specificity for nitrotyrosine, the antinitrotyrosine antibody was pretreated for at least 1 h at room temperature with 3-nitrotyrosine (Sigma, 10 mM solution, pH 7.4) prior to incubation on tissue sections. Immunohistochemical staining was assessed by two independent observers (KR, AJ) including a pathologist (KR).

### NADPH-diaphorase histochemistry

In total, 5 *μ*m cryostat sections were fixed for 15 min in a freshly made paraformaldehyde solution (4% w v^−1^ in PBS) and then washed four times in PBS. The NADPH-diaphorase (NADPH-d) activity of NOS, which is paraformaldehyde-resistant, was demonstrated by enzymatic reduction of nitroblue tetrazolium in the presence of NADPH, as previously described (Weinberg *et al*, 1996). Briefly, slides were incubated in 1 × PBS containing 1 mg ml^−1^ NADPH (Roche Molecular Biochemicals, Meylan, France) and 0.4 mg ml^−1^ NBT (Roche Molecular Biochemicals) for 30 min at 37°C, washed in PBS, dehydrated and mounted without any counterstaining. NADPH was omitted in negative controls.

### Quantitative RT–PCR analysis of NOS1 expression

Total RNA was extracted from four cases of Führman III RCC and normal renal parenchyma from the same patients using the GenElute Mammalian Total RNA kit (Sigma, Saint-Quentin Fallavier, France) and the Fast Prep cell disrupter (Bio 101, Q-BIOgene, Illkirch, France). RNA (5 *μ*g) was denaturated at 72°C for 3 min and then reverse transcribed for 60 min at 42°C in a 20 *μ*l reaction volume (50 mM Tris-HCl pH 8.3, 75 mM KCl, 3 mM MgCl_2_, 10 mM DTT) containing 0.5 *μ*g of random hexamers (Promega, Charbonnières, France), dNTPs (1 mM each), RNasin (50 U) and RnaseH^−^ MMLV reverse transcriptase (200 U).

The amplification conditions of the NOS1 and *β*-actin templates were optimised for the Rotorgene 2000 instrument (Ozyme, Saint Quentin en Yvelines, France). PCR amplifications were performed using Titanium Taq DNA polymerase (Clontech, Ozyme). The reaction mixture contained 2 *μ*l of the supplied 10 × Titanium Taq PCR buffer (containing magnesium chloride), 2 *μ*l of a 1/1000 dilution of SYBR Green I (Roche Molecular Biochemicals), 1 *μ*l of each primer (0.4 *μ*M each), 0.4 *μ*l of Titanium Taq DNA polymerase, 0.5 *μ*l of dNTPs (10 mM each) and PCR-grade water to a volume of 15 *μ*l. Microtubes (0.2 ml) were loaded with 15 *μ*l of this master mix and 5 *μ*l of the template (cDNA diluted 1/50) and the run was initiated. The cycling conditions were as follows: denaturation for 5 min at 95°C; amplification for 35 cycles, with denaturation for 5 s at 95°C, annealing for 10 s at 63°C and extension for 20 s at 72°C. To exclude primer-dimer artifacts, fluorescence was not measured at the end of the extension step, but a separate detection step was added (15 s) at a temperature (88°C) above the melting point of primer-dimers and below the melting point of the specific PCR product (92°C).

Primers NOS1 sense (5′-TCT CCT CCT ACT CTG ACT CC-3′) and NOS1 antisense (5′-TTG TGG ACA TTG GAT AGA CC-3′) were designed from the sequence of the human NOS1 cDNA (accession number NM-000620). They were selected for binding to separate exons (exon 17 and exon 20, respectively) to avoid false positive results arising from amplification of contaminating genomic DNA.

After completion of the cycling process, samples were subjected to a temperature ramp from 63 to 99°C, with continuous fluorescence monitoring for melting curve analysis. For each amplification, apart from primer-dimers, a single narrow peak was obtained at the expected melting temperature (92°C), indicating specific amplification without significant by-products.

An external standard curve was generated with serial five-fold dilutions of cDNA (1 : 20, 1 : 100 and 1 : 500) prepared from normal kidney RNA. The reference curve was constructed by plotting the relative amounts of these dilutions *vs* the corresponding Ct (threshold cycle) values. The correlation coefficient of these curves was always greater than 0.99. The amount of NOS1 transcript was calculated from these standard curves using the RotorGene software. A relative unit of 100 was assigned for the standard cDNA diluted 1/500. Samples were tested in triplicate and the average values were used for quantification.

For each sample, the relative amount of human *β*-actin cDNA was determined, as described for NOS1 with primers 5′-CCT TCC TGG GCA TGG AGT CCT G-3′ (actin sense) and 5′-GGA GCA ATG ATC TTG ATC TTC-3′ (actin antisense). The ratio between the relative amount of NOS1 and *β*-actin was then calculated to compensate for variations in quantity or quality of starting mRNA as well as for differences in reverse transcriptase efficiency.

## RESULTS

### Downregulation of NOS1 expression in high-grade clear cell RCC detected by immunohistochemistry, associated with a loss of the NADPH-diaphorase activity of NOS

We assessed the expression of NOS1 in tumour samples by using an antibody directed to the C-terminus part of NOS1 on cryostat sections. To confirm the staining pattern, we used another antibody directed to the N-terminus part of NOS1, on paraffin sections of a few cases of each type of tumour. The overall staining pattern was found identical with both antibodies (Table 2

**Table 2:** NADPH-diaphorase activity of NOS and NOS1 expression in renal tumours

	**NOS1 immunohistochemistry**		
**Tumour type**	**Cryostat sections (*n*=29)**	**Paraffinsections(*n*=9)**	**NADPH-d activity (*n*=29)**
Oncocytoma	5/5	2/2	5/5
Chromophobe RCC	2/2	2/2	2/2
*Clear cell RCC*			
Fürhman I	5/5	1/1	5/5
Fürhman II	1/7	1/2	1/7
Fürhman III	0/7	0/1	0/7
Fürhman IV	0/3	0/1	0/3

**Figure 1:**
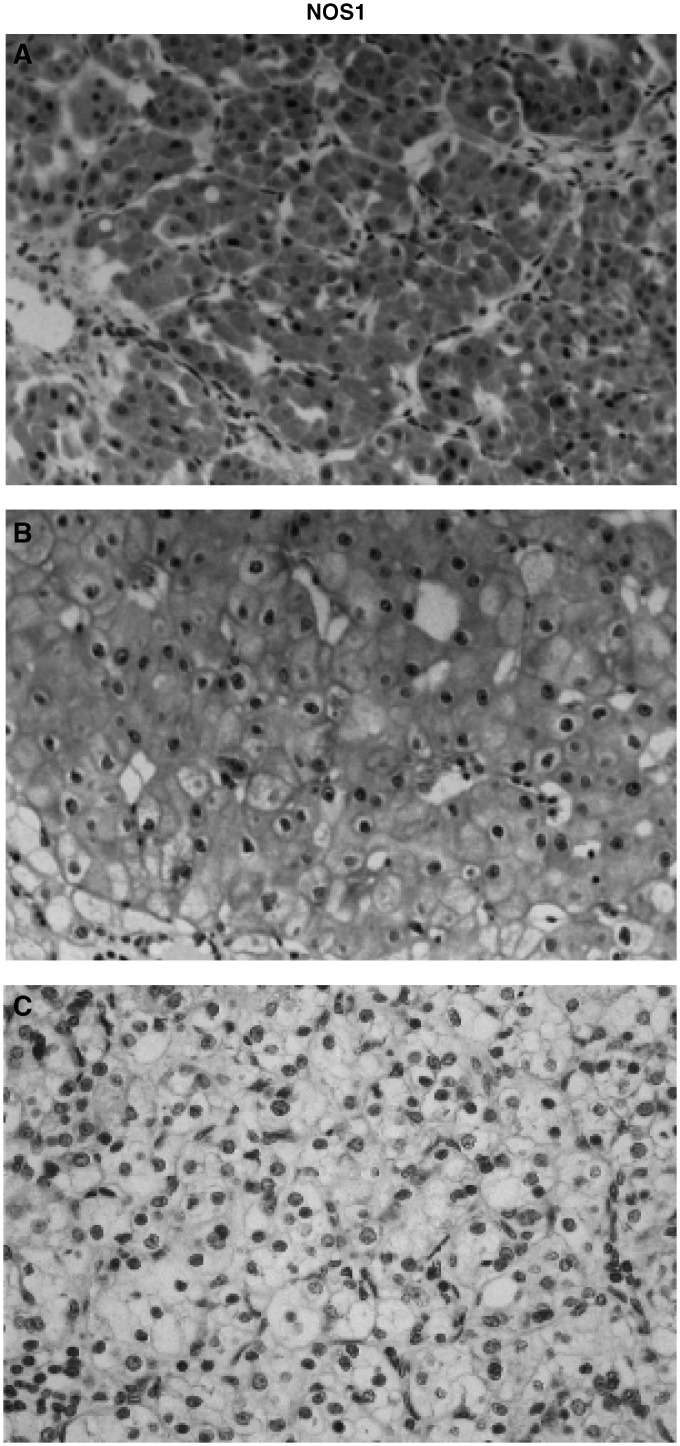
Correlation of NOS1 expression in human renal tumours with the tumour grade. Immunolocalisation of NOS1 on paraffin sections of a representative case of benign tumour (oncocytoma: **A**), low-malignant carcinoma (chromophobe RCC: **B**) and grade III clear cell RCC (**C**). Tumour cells were strongly labelled with the NOS1 antibody in the oncocytoma and chromophobe cell carcinoma, whereas they were negative in the high-grade RCC (original magnification × 200).

NADPH-diaphorase histochemistry was performed on cyrostat sections, and NOS1 immunohistochemistry on cryostat or paraffin sections depending on the antibody used (see Material and Methods). The numbers indicated in the table represent the number of positive tumours over the total number of tumours studied.

). NOS1 was expressed in tumour cells of oncocytomas, chromophobe RCC and in few cells of Fürhman I and II clear cell RCC. Conversely, Fürhman III and IV clear cell RCC never expressed NOS1 (Figure 1

).

We then confirmed that the downregulation of NOS1 immunoreactivity was in parallel with a loss of the NOS reductase activity. The results of the histochemical detection of NOS reductase activity by NADPH-d histochemistry, performed on paraformaldehyde-fixed cryostat sections of the different renal tumours, are summarised in Table 2. A high NADPH-d activity was detected in tumour cells of benign tumours and tumours of low malignancy, that is, oncocytoma and chromophobe RCC, respectively. A lower and heterogeneous staining pattern was observed in tumour cells of low-grade clear cell RCC (Fürhman grade I and rarely grade II). No activity was detected in Fürhman grade III or IV clear cell RCC. In all type of tumours, endothelial cells displayed a NADPH-d activity.

### The downregulation of NOS1 expression in high-grade clear cell RCC is correlated with a dramatic decrease in NOS1 mRNA levels

**Figure 2:**
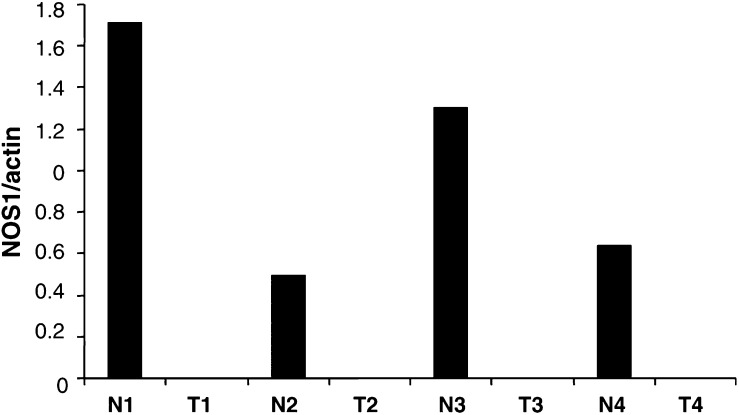
Quantitative analysis of NOS1 mRNA in grade III clear cell RCC. Total RNAs were extracted from the tumour of four patients undergoing surgery for grade III clear cell RCC (T1–T4) and also from the corresponding normal renal parenchyma (N1–N4) taken at distance from the tumour, reversed transcribed and submitted to real time PCR as described in Material and Methods. Amounts of NOS1 amplification products were normalised to *β*-actin. In the four cases, NOS1 mRNAs were beyond the limit of detection in the tumours whereas they were always present in the corresponding normal renal parenchyma.

In order to determine whether the lack of NOS1 expression was correlated with its mRNA level, the amount of NOS1 mRNA was measured by real-time quantitative RT–PCR in four specimens of grade III clear cell RCC and the corresponding normal tissue. As shown in Figure 2

, the level of NOS1 mRNAs relative to *β*-actin levels was undetectable in the tumour tissue of grade III clear cell RCC, whereas high NOS1 levels were found in the corresponding normal renal tissue.

### The downregulation of NOS1 expression in high-grade clear cell RCC is not associated with an upregulation of other NOS isoforms expression in tumour cells

To further confirm that the loss of NOS1 expression was not associated with an upregulation of other NOS isoforms, we assessed both NOS2 and NOS3 expression by immunohistochemistry in the renal tumours. In our study we did not find any NOS2 expression in tumour cells whatever the tumour type or grade. Only a few NOS2-positive inflammatory cells were noted within the stromal reaction and served as an internal positive control. NOS3 was never found in tumour cells, but, as expected from the NADPH-d assay, endothelial cells were stained with the NOS3 antibody.

### The downregulation of NOS1 expression in high-grade clear cell RCC is not associated with a loss of sGC, the physiological intracellular target of NO^•^

We assessed by immunohistochemistry the expression of sGC in two cases of each tumour type. The sGC was expressed in tumour cells of oncocytomas, chromophobe RCC and clear cell RCC whatever the Fürhman grade (not shown).

### Pattern of protein nitration in renal tumours compared with normal renal parenchyma

As protein tyrosine nitration is a post-translational modification of many proteins that can be accounted for by several mechanisms that is, formation of ONOO^−^ or other reactive nitrogen species (NO_2_^•^, NO_2_Cl) (Ischiropoulos, 1998; Wink and Mitchell, 1998), it was important to design a protocol to distinguish between these different processes. Interestingly, as the chemistry of ONOO^−^ is limited within close proximity of its source, only proteins located proximally to its site of generation can be nitrated. In contrast, other nitrating species, including the oxidation of NO_2_^−^ into NO_2_^•^ in the presence of myeloperoxidase or other haeme proteins (Grzelak *et al*, 2001) as well as the formation of NO_2_Cl, are known to have a long range of action and nitrate proteins at distance from their production site, that is, inflammatory cells (Wink and Mitchell, 1998; Espey *et al*, 2002). Since red blood cells are devoid of NOS, the assessment of red blood cells nitration can thus be considered as a simple index of the production in the cellular environment of reactive nitrogen species with a long range of action.

We assessed the pattern of protein nitration by immunohistochemistry in three cases of normal parenchyma and in three cases of each tumour type. In normal renal parenchyma the overall staining pattern was identical to the NOS1 expression pattern (Table 3

**Table 3:** Immunostaining pattern of protein nitration compared with that of NOS1 in the normal human renal parenchyma

	**Immunostaining pattern**	
**Intrarenal distribution**	**3-nitrotyrosine**	**NOS1^a^**
*Glomerulus*		
Mesangial cells	−	−
Parietal cells	±	−
Podocytes	±	±
		
Proximal tubules	+	+
Distal tubules	+	+
Henle's loops	+	+
Collecting ducts	+	+
Red blood cells	−	−

3-Nitrotyrosine immunohistochemistry was performed on paraffin section of three cases of normal renal parenchyma.

−=no staining; ±=heterogeneous positive staining; +=homogeneous positive staining.

a NOS1 staining pattern already fully described in normal kidney in a previous paper (Jarry *et al*, 2003).

and Figure 3A

**Figure 3:**
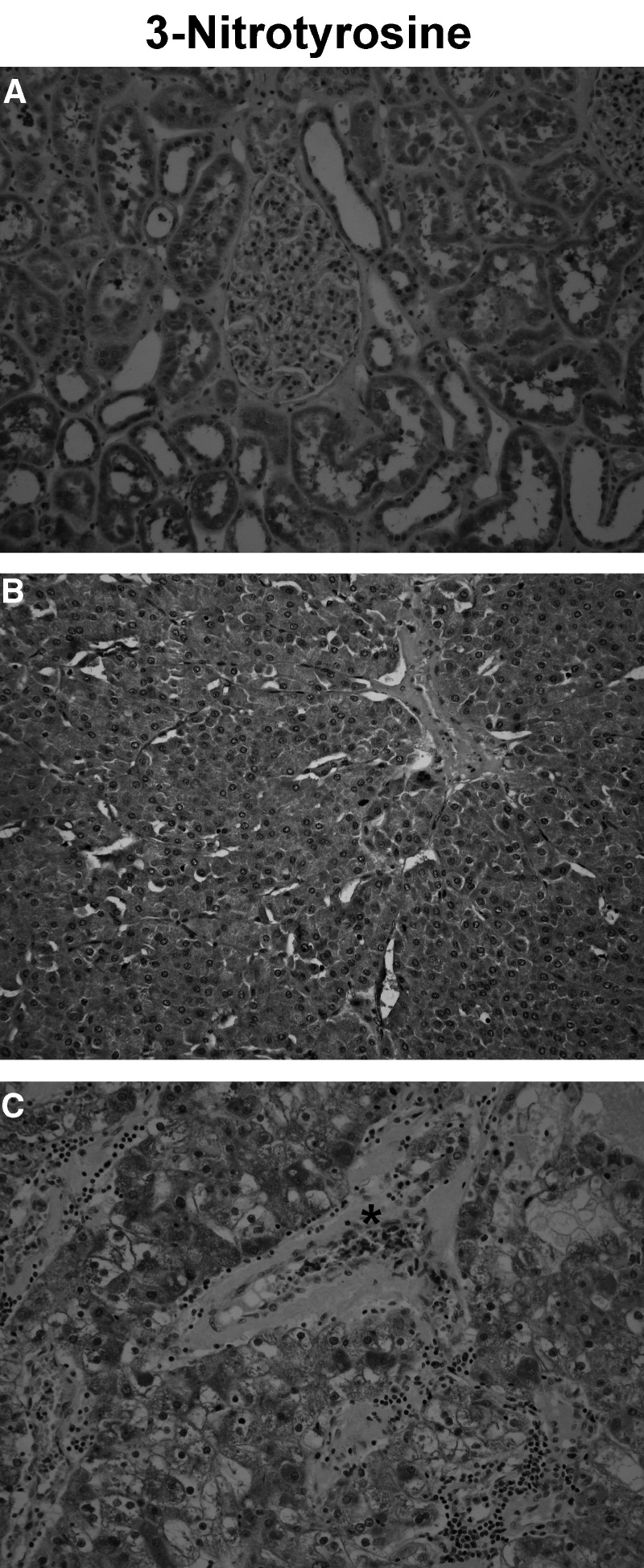
Changes in the immunostaining pattern of protein tyrosine nitration between normal human kidney/benign tumour and RCC. Immunodetection of 3-nitrotyrosine on paraffin sections. (**A**) In normal renal parenchyma, most of the tubular epithelial cells along the nephron were labelled, with a variable staining intensity. In glomeruli, some of the podocytes and parietal cells scored positive. Red blood cells were not labelled. (**B**) In oncocytoma, a strong, granular and homogeneous staining of tumour cells was noted. Red blood cells were not labelled, and there was no inflammatory infiltrate. (**C**) In contrast, in malignant tumours (a case of grade III clear cell RCC is shown), the pattern of protein tyrosine nitration was heterogeneous. Red blood cells (^*^) were strongly stained and an inflammatory infiltrate was present (original magnification × 200).

), with no red blood cell nitration. In benign tumours (i.e. oncocytoma), the staining pattern of nitration was similar to the one of NOS1 with a strong, granular and homogeneous staining of tumour cells, and without any staining of red blood cells in the absence of inflammatory infiltrate (Table 4

**Table 4:** Pattern of protein nitration in renal tumours: correlation with the nitration of red blood cells and the degree of the inflammatory infiltrate

	**3-Nitrotyrosine immunohistochemistry**		
**Tumour type**	**Tumour cells**	**Red blood cells**	**Inflammatory infiltrate**
Oncocytoma	Homogeneous	−	−
Chromophobe RCC	Heterogeneous	+	+
*Clear cell RCC*			
Fürhman I	Heterogeneous	+	++
Fürhman II	Heterogeneous	+	+++
Fürhman III	Heterogeneous	+	+++
Fürhman IV	Heterogeneous	+	+++

3-Nitrotyrosine immunohistochemistry was performed on paraffin sections of three cases of each tumour type. The immunostaining of tumour cells was referred to as ‘homogeneous’ when a majority of cells was stained with the same intensity and as ‘heterogeneous’ when some parts of the tumour were stained and others devoid of any staining. Immunostaining of red blood cells: (−) denotes absent and (+) denotes majority of red blood cells stained.

Semiquantitative evaluation of the inflammatory infiltrate: (−) means absence of infiltrate; (+) denotes a mild infiltrate, (++) a moderate infiltrate and (+++) an abundant infiltrate.

and Figure 3B). In contrast, in malignant tumours (i.e. chromophobe RCC and clear cell RCC), surrounded by an important inflammatory infiltrate, the pattern of protein nitration was highly heterogeneous, with some parts of the tumour strongly stained and others devoid of any staining. In addition, there was an intense staining of red blood cells in vessels within or near the tumour (Table 4 and Figure 3C).

## DISCUSSION

Our results show a modulation of NOS1 expression correlating with the pathological grading and the malignant potential of renal tumours. These results support and extend previous findings showing that Ca^2+^-dependent NOS activity, measured by L-arginine to L-citrulline conversion assay, was significantly lower in RCCs than in normal renal parenchyma and inversely correlated with the tumour grade according to Führman's classification (Jansson *et al*, 1998). In this study the calcium-independent inducible NOS activity was undetectable in RCC (Jansson *et al*, 1998). Similarly, in our study, NOS2 was never detected by immunohistochemistry in all the tumours tested, thus suggesting that the loss of NOS1 expression was not counterbalanced by induction of NOS2 expression in tumour cells. The downregulation of NOS1 expression in high-grade clear cell RCC is associated with the absence of NOS1 mRNA, as shown by real-time RT–PCR. Numerous splicing variants of the *NOS1* gene have been described (Forstermann *et al*, 1998). Most of them differ in their 5′ end. One cannot exclude that high-grade clear cell RCC tumour cells express a specific splicing variant of NOS1 mRNA, which would not have been detected in our experimental conditions. But this is unlikely, since the PCR primers were designed to span a large central area of the transcript. Furthermore, we used on purpose two antibodies specific for the N- and C-terminus part of NOS1, which should have allowed us to detect a truncated protein. Therefore, we can conclude that the absence of detectable NOS1 mRNA is probably due to a significant decrease in *NOS1* gene transcription or/and in NOS1 mRNA stability, although a deletion or a mutation in the *NOS1* gene cannot yet be excluded. Further investigations are needed to elucidate this issue.

Another interesting finding of our study is that the loss of NOS1 expression in high-grade RCC is not associated with a loss of sGC expression. This result leads to two conclusions: (i) the autocrine signalling pathway of NO previously observed in normal human tubular epithelial cells (Jarry *et al*, 2003) is maintained in benign tumours and low malignant potential RCC but not in high-grade clear cell RCC, and (ii) the persistence of sGC expression in all tumour types implies that tumour cells remain sensitive to the bioregulatory roles of exogeneous NO.

The third major finding of this study concerns the pattern of protein tyrosine nitration, which is identical in normal kidney and in benign renal tumours, but completely different in malignant tumours. Nitration is a covalent modification of the tyrosine residues of some proteins with activating or inhibitory activities that could be involved in physiological conditions as well as in pathological states such as inflammatory processes and tumours (Aulak *et al*, 2001; Greenacre and Ischiropoulos 2001; Ehsan *et al*, 2002; Ischiropoulos, 2003; Schopfer *et al*, 2003). Nitration process can result from several mechanisms, that is, intracellular action of ONOO^−^ and long-range action of other reactive nitrogen species produced by inflammatory cells. The most well-known mechanism of protein nitration is the ONOO^−^ pathway, resulting from the reaction of equimolar NO^•^ and O_2_^−^, a phenomenon restricted to an organelle or cellular compartment such as mitochondria (Radi *et al*, 2002; Ghafourifar and Colton, 2003). Our observation of a granular staining pattern with the antinitrotyrosine antibody, identical to that of NOS1, in tubular epithelial cells of normal renal parenchyma and in oncocytoma, is in line with this concept of ONOO^−^ chemistry limited to close proximity of its source. In addition, we can rule out the involvement of long-range acting nitrogen species since red blood cells are devoid of any staining in normal renal tissue and in oncocytoma. In malignant tumours containing an important inflammatory infiltrate, the interpretation of protein nitration pattern is more complex. Indeed, other nitrating species known to have a long range of action are produced by inflammatory cells and result from the oxidation of circulating nitrite NO_2_^−^ into NO_2_^•^, a reaction catalysed by myeloperoxidase or other haeme proteins in the presence of H_2_O_2_ (Wink and Mitchell, 1998). In addition, the Cl^−^ transfer from HOCl to NO_2_^−^ generates NO_2_Cl, a nitrating agent (Bian *et al*, 2003). Since we noticed a downregulation of NOS1 expression in most malignant renal tumours, we can suggest that the reactive nitrogen species released by the inflammatory infiltrate are responsible for the heterogeneous nitrated staining pattern observed in these malignant tumours. This interpretation was strongly supported by the staining of red blood cells, a cell type devoid of any endogeneous source of NO.

In conclusion, our study helped decipher the pattern of NO signalling in renal tumours of different grades compared with that of normal renal parenchyma. The bioregulatory activity of NO is similar in benign tumours and in normal kidney, whereas there is a shift of NO signalling in high-grade malignant tumours, with a loss of endogeneous NO synthesis, but the maintenance of sGC, the receptor for exogeneous NO. The demonstration of nitrated proteins in renal tumours implies that exogeneous NO, especially reactive nitrogen species produced by inflammatory cells, have an impact on cancer cells, whose biological significance remain to be determined.
